# The cost of comorbidities in treatment for HIV/AIDS in California

**DOI:** 10.1371/journal.pone.0189392

**Published:** 2017-12-14

**Authors:** David S. Zingmond, Kodi B. Arfer, Jennifer L. Gildner, Arleen A. Leibowitz

**Affiliations:** 1 Division of General Internal Medicine and Health Services Research, The David Geffen School of Medicine at UCLA, Los Angeles, California, United States of America; 2 Department Medicine, Veterans Affairs Greater Los Angeles Healthcare System (GLA), Los Angeles, California, United States of America; 3 Global Center for Children and Families, UCLA, Los Angeles, California, United States of America; 4 Department of Public Policy, UCLA, Los Angeles, California, United States of America; Katholieke Universiteit Leuven Rega Institute for Medical Research, BELGIUM

## Abstract

**Background:**

Antiretroviral therapy has increased longevity for people living with HIV (PLWH). As a result, PLWH increasingly experience the common diseases of aging and the resources needed to manage these comorbidities are increasing. This paper characterizes the number and types of comorbidities diagnosed among PLWH covered by Medicare and examines how non-HIV comorbidities relate to outpatient, inpatient, and pharmaceutical expenditures.

**Methods:**

The study examined Medicare expenditures for 9767 HIV-positive Californians enrolled in Medicare in 2010 (7208 persons dually covered by Medicare and Medicaid and 2559 with Medicare only). Costs included both out of pocket costs and those paid by Medicare and Medicaid. Comorbidities were determined by examining diagnosis codes.

**Findings:**

Medicare expenditures for Californians with HIV averaged $47,036 in 2010, with drugs accounting for about 2/3 of the total and outpatient costs 19% of the total. Inpatient costs accounted for 18% of the total. About 64% of the sample had at least one comorbidity in addition to HIV. Cross-validation showed that adding information on comorbidities to the quantile regression improved the accuracy of predicted individual expenditures. Non-HIV comorbidities relating to health habits—diabetes, hypertension, liver disease (hepatitis C), renal insufficiency—are common among PLWH. Cancer was relatively rare, but added significantly to cost. Comorbidities had little effect on pharmaceutical costs, which were dominated by the cost of antiretroviral therapy, but had a major effect on hospital admission.

**Conclusions:**

Comorbidities are prevalent among PLWH and add substantially to treatment costs for PLWH. Many of these comorbidities relate to health habits that could be addressed with additional prevention in ambulatory care, thereby improving health outcomes and ultimately reducing costs.

## Introduction

Care for people infected with HIV has traditionally focused on optimal management of HIV infection and ensuring access to providers with specialty knowledge of HIV care. The success in managing HIV infection has led to increased survival for people living with HIV (PLWH), with the result that in 2014 45% of Americans diagnosed with HIV were aged 50 and older.[1] As a result, PLWH are increasingly experiencing the common diseases of aging.

Non-HIV comorbidities are common among those infected with HIV, particularly among Medicare and Medicaid enrollees. [2–12] The prevalence of comorbidities rises with age. An analysis of health insurance claims data found that 83% of PLWH aged 50 and older and 63% of PLWH aged 18–49 had at least one comorbidity. In contrast, 69% of age/gender matched controls without HIV who were 50 and older had at least one medical condition.[13] A study of Medicare enrollees with HIV found that 20.1% had kidney disease, 19.4% had diabetes, and 27.3% had hyperlipidemia, and 16.0% experienced a cardiovascular event in 2013.[2] Cancer is more prevalent among PLWH than in the general population.[14] Among Medicare enrollees over 65, those with HIV were diagnosed with new cancers over a 5 year period at a rate 50% greater than the cancer incidence rate for Medicare enrollees without HIV.[15]

Multimorbidity is also common among PLWH. Insurance claims data show that among PLWH aged 50 and older, 22% had one medical condition in addition to HIV, 36% had 2–3 comorbidities, 15% had 4–5, and 10% had >5.[13] There is also evidence that PLWH may develop some diseases at earlier ages than people who are not infected with HIV due to immune exhaustion from chronic inflammation engendered by even well-controlled/treated HIV infection.[16]. A study in Italy estimated that PLWH experience rates of non-infectious comorbidities that were comparable to those of patients without HIV who were 10–15 years older. [11]

As care for HIV infections has matured and PLWH are living longer without AIDS, the resources needed to manage and treat their non-HIV comorbidities have increased in importance. One measure of these resources is the cost of treating these comorbidities, which adds to the substantial costs of HIV/AIDS treatment. Historically, overall costs of HIV treatment have been driven largely by the high costs of antiretrovirals and of hospitalizations.[17,18] In recent guidelines for use of ART, HHS calls on providers to inform themselves about antiretroviral regimen costs, patient insurance regulations, and availability of antiretroviral therapy (ART).[19] While the costs of comorbidities have been examined for PLWH in Italy and in Canada, little is known about how treatment for non-HIV comorbidities relate to outpatient, inpatient and pharmaceutical costs for PLWH in the United States, particularly those who are publicly insured.[10–12]

This paper seeks, first, to characterize the number and types of comorbidities diagnosed among publicly-insured PLWH covered by Medicare. Secondly, we examine how comorbidities predict outpatient, inpatient and pharmaceutical expenditure.

## Materials and methods

### Data

Insurance claims data for HIV-positive Californians enrolled in Medicare in 2010 were acquired through a confidential data use agreement with the Center for Medicare and Medicaid Services (CMS). The sample includes Medicare beneficiaries enrolled in both Medicare (the primary payer) and Medicaid (hereafter, Duals) as well as those enrolled solely in Medicare (Medicare-only). We applied a case-identification algorithm to create an analysis file of adult beneficiaries with verifiable HIV. [20] Our analyses were limited to fee-for-service Medicare enrollees because available data for Medicare managed care enrollees lack diagnosis fields needed to confirm HIV status and identify comorbidities. In order to account for medication expenditures, we required enrollment in Part D drug coverage for the entire year of 2010. There were 7208 Californians with HIV who met the sample inclusion criteria and were dually covered by Medicare and Medicaid and 2559 Californians who had only Medicare coverage.

Data acquisition was reviewed and approved by the CMS Privacy Board. The UCLA Institutional Review Board (UCLA IRB #10–000823) reviewed the study and deemed it exempt. Data were obtained in research identifiable files; storage, analysis, and reporting met CMS data security requirements.

### Outcome variables

This analysis separately examines health care expenditures for outpatient services, pharmaceuticals, and inpatient services. Expenditures included all costs reimbursed by Medicare and Medicaid plus costs paid by the patient (e.g. deductible and coinsurance) in each category. Thus pharmaceutical costs include patients’ payments in the “doughnut hole” phase, where Medicare does not reimburse for drug purchases.

### Comorbidities

Diagnoses were grouped using the typology of 26 comorbidities developed by Elixhauser.[21,22] An indicator variable for each comorbidity was set to one if an individual’s medical claims contained an ICD-9 diagnosis code at least once in 2010 in an inpatient claim or at least twice in outpatient claims on different days. For each individual, the number of unique comorbidities was calculated. AIDS-defining conditions were not included among comorbidities and hemophilia was treated separately as a subgroup of coagulopathy due to extremely high costs. CMS has redacted substance use diagnoses from the public use files, therefore we could not include substance use as a comorbidity.

### Individual characteristics

CMS enrollment data provided information on participant age, race, and gender. Reason for Medicare coverage was coded as “disability” or other (e.g. aged). RUCA coding was used to determine urban or rural designation based on participant’s zip code. An individual’s access to an HIV specialist was indicated by whether they had an evaluation and management visit with any provider who treated 50 or more Medicare or Medicaid HIV patients in California in 2010.

### Statistical analysis

Mean and median medical costs were calculated by number of comorbidities. Multi-variable methods were used to examine the effect of a given chronic disease on median costs, net of other comorbidities and demographic factors. We use quantile regression to examine median expenditures, where the goal is to minimize the absolute difference between actual and predicted costs. In order to examine the percentage increase in medical costs, we made a logarithmic transformation of each individual’s expenditure. Analyses were carried out using *R* in the quantreg package [23]

More than 99% of the sample used outpatient care and had pharmaceutical claims in 2010. In contrast, only a minority of patients were hospitalized in 2010 (20% of Duals and 13% of Medicare-only). Therefore, we used logistic regression to relate each comorbidity to the probability of hospitalization, holding demographic factors and other comorbidities constant.

We fit quantile-regression models to estimate the relationship between the demographic and comorbidity variables and the median logged outpatient and drug spending.[23] Inpatient expenditures were modeled among patients who were hospitalized at all. Age and age squared, standardized to have mean 0 and standard deviation 1/2, were included to allow for the quadratic effect of age.[24] The resulting coefficients indicate the percentage change in the median associated with each independent variable.

We estimated a variety of alternative models and used cross-validation to select the model that minimized the mean absolute error (MAE) between the predicted and the observed values for outpatient and drug costs. By assessing the models in terms of MAE, rather than root mean square error (RMSE), we obtain a cost prediction for the typical patient. In contrast, predictions from estimating models that minimize RMSE tend to overestimate costs for the majority of patients in order to reduce prediction errors for the small percent of very high cost patients.

We compared observed costs at the individual level to costs predicted by models with no covariates, only demographic variables and with both demographic and comorbidity measures. This provided a test of whether comorbidities have predictive value beyond that of the demographic variables alone. For outpatient and drug costs we examined whether separate models for Medicare-only beneficiaries and dual beneficiaries predicted costs better than a single model that included a dummy variable distinguishing these beneficiary types. Thus, we compared 6 models (3 sets of independent variables for both separate and combined modeling of beneficiary type).

Inpatient costs were estimated with a two-step procedure. A logistic-regression predicted the probability of having nonzero inpatient costs and a quantile-regression predicted the median expenditure, conditional on having nonzero inpatient costs. The 9 models we compared crossed 3 different sets of independent variables for the logistic model with the same three sets for the quantile regression model.

We assessed each model with tenfold cross-validation by randomly assigning patients to one of ten folds (subsamples), then predicting costs for each patient in each fold. We then calculated the mean absolute error (MAE) between the predicted and observed cost for each patient We assessed prediction accuracy based on the mean of these MAEs.

## Results

### Sociodemographics

The majority of this sample of PLWH who are enrolled in Medicare are male (90%) and between the ages of 41 and 60 (72%). (Table 1). Whites comprise 57% of the sample while African Americans account for 19% and Hispanics 20%. Over 94% of the sample live in urban areas and approximately two-thirds (63%) had access to a provider treating 50 or more publicly insured patients. Seventy four percent of the Medicare sample also have Medicaid coverage and most (91%) qualified for Medicare due to long-term disability.

**Table 1:** Prevalence, mean and median annual costs by demographics, number of comorbidities, and spending type (N = 9767).

		%	Mean Spending	Median Spending
Enrollment	Medicare Only	26.2%	$41,606	$32,573
	Dual	73.8%	$48,964	$35,051
Sex	Male	89.3%	$47,010	$34,381
	Female	10.7%	$47,251	$34,249
Race	White	56.9%	$46,260	$34,791
	African American	19.1%	$51,062	$34,340
	Hispanic	19.7%	$45,208	$33,453
	Other	4.3%	$47,821	$34,038
Age category	< = 40	9.3%	$43,167	$29,330
	41–50	38.2%	$46,206	$33,968
	51–60	33.4%	$48,124	$36,417
	>60	19.0%	$48,697	$34,142
Eligibility	Other	9.4%	$47,184	$32,796
	Disabled	90.6%	$47,021	$34,549
Area	Rural	5.1%	$43,068	$32,676
	Urban	94.9%	$47,251	$34,467
Highest volume HIV provider	0–49 patients	37.2%	$48,933	$34,202
	50+ patients	62.8%	$45,911	$34,485
Total spending			$47,036	$34,379
	Outpatient	99.8%	$8,714	$4,085
	Drug	99.3%	$30,052	$26,633
	Inpatient	26.0%	$32,668	$13,487
Number of comorbidities	0	36.1%	$30,312	$27,795
	1	25.4%	$38,260	$32,949
	2	15.3%	$46,108	$37,371
	3	8.6%	$55,492	$44,865
	4	4.6%	$66,330	$52,515
	5	3.2%	$80,047	$59,992
	6	2.1%	$100,861	$81,006
	7	1.6%	$114,451	$87,323
	8–10	2.2%	$135,808	$116,811
	11 or more	0.9%	$218,940	$203,571

Means and medians for outpatient, drug, and inpatient spending are based on non-zero spending for the respective category.

The mean Medicare expenditure for California enrollees with HIV was $47,036 in 2010 (Table 1). Drug costs were the largest expense category, accounting for almost 2/3 of the total ($30,052). Mean outpatient costs of $8,714 amounted to 19% of total annual expenditures. The 26% of the sample who experienced an inpatient episode had mean hospital costs of $32,668. Across the entire sample, inpatient costs accounted for 18% of total costs for the average patient.

### Comorbidities

Comorbidities were common (Table 1). About 64% of this HIV-positive sample had at least one additional condition. Both mean and median per capita treatment costs rose monotonically with the number of comorbidities.

Table 2 shows the prevalence of individual comorbidities among sample members. The most frequently identified comorbidity was uncomplicated hypertension (32%). A diagnosis of complicated hypertension was received by 5% of enrollees. Uncomplicated diabetes, liver disease, and chronic pulmonary disease were each diagnosed in more than 10% of the sample. The prevalence of cancer was relatively rare: 2% of the sample had lymphoma, fewer than 1% had metastatic cancer and 6% had solid tumors without metastasis.

**Table 2:** Prevalence, mean and median spending by comorbidities (N = 9767).

Comorbidities	%	Mean Spending	Median Spending
Congestive heart failure	5.3%	$104,641	$76,152
Cardiac arrhythmias	6.7%	$100,330	$67,335
Valvular disease	2.7%	$108,204	$72,559
Peripheral vascular disorders	4.7%	$89,102	$59,363
Hypertension, uncomplicated	31.8%	$62,231	$42,183
Hypertension, complicated	5.4%	$108,442	$79,604
Paralysis	1.1%	$112,051	$73,815
Other neurological disorders	5.3%	$97,511	$64,454
Pulmonary circulation disorders	1.5%	$119,181	$91,819
Chronic pulmonary disease	13.7%	$74,391	$50,919
Diabetes, uncomplicated	14.4%	$66,275	$45,330
Diabetes, complicated	4.4%	$92,992	$62,640
Hypothyroidism	6.2%	$69,416	$50,998
Renal failure	9.0%	$88,320	$60,545
Liver disease	15.8%	$68,169	$43,903
Peptic ulcer disease	0.7%	$110,610	$66,769
Lymphoma	1.8%	$73,641	$49,466
Metastatic cancer	0.6%	$117,362	$91,779
Solid tumor without metastasis	6.0%	$67,164	$49,879
Rheumatoid arthritis	2.3%	$74,563	$48,873
Coagulopathy	3.2%	$109,681	$77,177
Coagulopathy—hemophilia	0.3%	$369,119	$227,917
Blood loss anemia	0.7%	$128,091	$101,333
Deficiency anemia	6.9%	$90,258	$60,130
Obesity	2.9%	$65,049	$48,611
Weight loss	7.6%	$97,346	$65,176
Fluid and electrolyte disorders	9.7%	$107,681	$77,998

Mean and median per capita total expenditures for individuals with each comorbidity group are also shown in Table 2. Costs varied substantially across diagnoses. There is a negative correlation of -0.5 between prevalence and cost (p < .05), with the least frequent comorbidities (like hemophilia) averaging very high per capita costs, as compared to the more prevalent comorbidities such as hypertension.

### Regression results

An individual can have more than one comorbidity, thus we examine net costs of each comorbidity in a multi-variable framework that controls for the presence of other comorbidities as well as demographic factors.

Quantile regression was used to relate logged expenditures for each of the cost components (outpatient, drugs, and inpatient costs, if hospitalized) to demographic factors and presence of comorbidities. (Table 3). Logistic regressions modeled the probability of inpatient use. (Table 4). Models that combined Medicare-Only and Dual samples, and included all demographic and comorbidity variables as predictors minimized MAE for outpatient and drug costs, although the improvement over the estimate without covariates was small. (Table 5). The improvement in prediction was greatest for the inpatient model, where adding comorbidities reduced the MAE by 20%, from $8,489 TO $6,792. (Table 6).

**Table 3:** Effect of comorbidities on outpatient, drug, and inpatient expenditures (percentage increase over median costs).

		Outpatient Cost (n = 9767)	Drug Cost (n = 9767)	Conditional Inpatient Cost (n = 2538)
		Coefficient Estimate (95% CI)	Coefficient Estimate (95% CI)	Coefficient Estimate (95% CI)
Intercept		2,151 (1804, 2405)	21,982 (20,522,23,625)	3,697 (2321,5561)
Enrollment	Dual	1.21 (1.15, 1.28)	1.04 (1.02, 1.08)	1.02 (0.80, 1.28)
Gender	Female	1.19 (1.11, 1.29)	0.89 (0.86, 0.92)	0.93 (0.74, 1.09)
Age	Linear	1.04 (0.98, 1.10)	1.04 (1.01, 1.07)	0.87 (0.71, 1.00)
	Quadratic	1.04 (0.97, 1.09)	0.85 (0.83, 0.89)	1.07 (0.91, 1.20)
Race	African American	0.84 (0.79, 0.89)	0.88 (0.85, 0.91)	1.04 (0.91, 1.28)
	Hispanic	0.84 (0.79, 0.88)	0.95 (0.92, 0.99)	0.98 (0.83, 1.27)
	Other	0.85 (0.75, 0.94)	0.99 (0.93, 1.04)	1.05 (0.69, 1.51)
Area	Urban	1.00 (0.93, 1.15)	1.05 (0.99, 1.10)	0.98 (0.74, 1.25)
Highest vol. HIV provider	50+ patients	1.11 (1.05, 1.16)	1.08 (1.05, 1.10)	0.90 (0.78, 1.07)
Eligibility	Disabled	1.05 (0.93, 1.17)	1.04 (0.98, 1.10)	1.17 (0.78, 1.67)
Comorbidities	Congestive heart failure	1.26 (1.13, 1.37)	0.95 (0.88, 1.02)	1.23 (1.00, 1.52)
	Cardiac arrhythmias	1.37 (1.22, 1.50)	1.01 (0.96, 1.07)	1.45 (1.26, 1.74)
	Valvular disease	1.27 (1.10, 1.44)	1.02 (0.94, 1.12)	1.08 (0.85, 1.41)
	Peripheral vascular disorder	1.33 (1.21, 1.52)	1.05 (0.98, 1.12)	1.19 (0.92, 1.51)
	Hypertension, uncomplicated	1.22 (1.17, 1.28)	1.06 (1.03, 1.09)	1.35 (1.14, 1.64)
	Hypertension, complicated	1.41 (1.26, 1.61)	0.91 (0.85, 0.98)	1.14 (0.94, 1.50)
	Paralysis	1.64 (1.31, 2.02)	1.01 (0.83, 1.18)	2.02 (1.45, 2.74)
	Other neurological disorders	1.40 (1.24, 1.53)	1.04 (0.98, 1.11)	1.66 (1.44, 2.09)
	Pulmonary circulat. disord.	1.01 (0.85, 1.37)	1.16 (1.07, 1.35)	1.25 (0.96, 1.75)
	Chronic pulmonary disease	1.36 (1.30, 1.46)	1.07 (1.03, 1.10)	1.37 (1.20, 1.65)
	Diabetes, uncomplicated	1.14 (1.07, 1.23)	1.10 (1.07, 1.15)	1.14 (0.89, 1.33)
	Diabetes, complicated	1.26 (1.12, 1.47)	0.94 (0.86, 1.03)	1.04 (0.85, 1.40)
	Hypothyroidism	1.33 (1.22, 1.44)	1.12 (1.07, 1.19)	1.08 (0.84, 1.33)
	Renal failure	1.40 (1.29, 1.51)	1.07 (1.02, 1.11)	1.25 (0.94, 1.50)
	Liver disease	1.41 (1.32, 1.50)	1.04 (1.00, 1.07)	1.40 (1.16, 1.64)
	Peptic ulcer disease	1.32 (1.00, 1.79)	0.88 (0.61, 1.06)	1.58 (1.15, 2.22)
	Lymphoma	1.68 (1.43, 1.88)	0.99 (0.91, 1.07)	1.53 (1.16, 2.05)
	Metastatic cancer	2.25 (1.41, 2.86)	1.05 (0.90, 1.18)	1.66 (1.07, 2.95)
	Solid tumor without metastasis	1.76 (1.56, 1.92)	1.05 (1.00, 1.11)	1.20 (0.91, 1.52)
	Rheumatoid arthritis	1.27 (1.11, 1.49)	1.00 (0.88, 1.11)	0.81 (0.58, 1.21)
	Coagulopathy	1.13 (1.01, 1.35)	0.97 (0.88, 1.04)	1.50 (1.19, 1.84)
	Hemophilia	20.39 (3.07, 40.03)	1.25 (1.00, 1.68)	2.28 (1.00, 4.29)
	Blood loss anemia	1.13 (0.93, 1.35)	0.99 (0.77, 1.26)	0.90 (0.73, 1.81)
	Deficiency anemia	1.66 (1.53, 1.81)	1.08 (1.01, 1.12)	1.24 (1.00, 1.44)
	Obesity	1.12 (1.03, 1.26)	0.98 (0.92, 1.07)	1.56 (1.11, 2.01)
	Weight loss	1.37 (1.25, 1.50)	1.05 (1.00, 1.12)	1.72 (1.40, 1.95)
	Fluid and electrolyte disord.	1.17 (1.07, 1.27)	0.94 (0.89, 1.00)	2.31 (2.01, 2.67)

**Table 4:** Odds ratios and 95% confidence intervals from logistic regression of probability of hospitalization on demographics and comorbidities.

		Odds Ratio (95% CI)
Enrollment	Dual	1.49 (1.29, 1.73)
Gender	Female	1.10 (0.91, 1.32)
Age	Linear	0.59 (0.51, 0.69)
	Quadratic	1.03 (0.90, 1.19)
Race	African American	1.00 (0.86, 1.17)
	Hispanic	0.96 (0.83, 1.12)
	Other	0.83 (0.61, 1.11)
Area	Urban	1.48 (1.12, 1.97)
Highest volume HIV provider	50+ patients	0.92 (0.82, 1.04)
Eligibility	Disabled	0.81 (0.60, 1.10)
Comorbidities	Congestive heart failure	1.50 (1.13, 1.99)
	Cardiac arrhythmias	3.85 (3.04, 4.87)
	Valvular disease	1.88 (1.28, 2.75)
	Peripheral vascular disorder	1.32 (1.00, 1.72)
	Hypertension, uncomplicated	1.87 (1.65, 2.13)
	Hypertension, complicated	2.81 (2.08, 3.80)
	Paralysis	4.79 (2.85, 8.20)
	Other neurological disorders	3.86 (3.02, 4.92)
	Pulmonary circulation disorders	2.24 (1.36, 3.71)
	Chronic pulmonary disease	2.65 (2.27, 3.09)
	Diabetes, uncomplicated	1.02 (0.85, 1.22)
	Diabetes, complicated	1.09 (0.79, 1.49)
	Hypothyroidism	1.21 (0.96, 1.52)
	Renal failure	0.93 (0.74, 1.18)
	Liver disease	1.76 (1.52, 2.04)
	Peptic ulcer disease	2.13 (1.01, 4.49)
	Lymphoma	2.63 (1.79, 3.86)
	Metastatic cancer	1.77 (0.82, 3.86)
	Solid tumor without metastasis	2.01 (1.60, 2.51)
	Rheumatoid arthritis	1.29 (0.89, 1.86)
	Coagulopathy	4.73 (3.36, 6.70)
	Hemophilia	0.84 (0.29, 2.22)
	Blood loss anemia	3.66 (1.74, 7.95)
	Deficiency anemia	1.19 (0.94, 1.50)
	Obesity	1.81 (1.33, 2.46)
	Weight loss	2.10 (1.70, 2.58)
	Fluid and electrolyte disorders	9.89 (7.99, 12.30)

**Table 5:** Effect of covariates on Mean Absolute Errors (MAE) of predictions of outpatient expenditures and drugs.

	No Controls	Demographic Controls	Comorbidity Controls
Outpatient	$6,555	$6,487	$6165
Drugs	$12,932	$12,683	$12,595

**Table 6:** Effect of covariates on Mean Absolute Errors (MAE) of predictions of inpatient expenditures.

Independent Variables Included in Inpatient Probability Regression	Independent Variables Included in Conditional Inpatient Regression		
	No Controls	Demographic Controls	Comorbidity Controls
No Controls	$8,489	$8489	$9832
Demographics	$8487	$8,505	$8,501
Comorbidities	$7,809	$7,777	$6,792

Each comorbidity coefficient can be interpreted as the percentage increase in median spending associated with this comorbidity compared to a non-disabled, white, urban, male Medicare-only enrollee of mean age with no other chronic conditions, who did not visit a high volume provider (50+ HIV patients).

Every comorbidity examined increased the cost of outpatient treatment. While obesity and uncomplicated diabetes had modest effects on outpatient costs, (12% and 14% increase in median costs, respectively), all three cancers added substantially to outpatient costs. In contrast, pharmaceutical expenses were relatively insensitive to individual conditions, except in the case of hemophilia.

The median inpatient cost differential associated with each comorbidity varied over a wide range. Logistic regressions (Table 4) show that, as expected, comorbidities and the likelihood of hospitalization are positively related. However, after controlling for comorbidities, age and Dual status had the greatest effects on the odds of hospitalization.

Median outpatient costs also varied by patients’ demographic characteristics. Hispanic PLWH incurred lower median cost for outpatient services, while African-Americans had lower costs for both outpatient services and drugs. Female PLWH had about 19% higher outpatient costs, but their medication costs were only 89% of males’. Age did not have a large effect, net of other demographics and comorbidities. Dual enrollees, with both Medicare and Medicaid coverage, had median outpatient costs that were 21% greater than Medicare-Only patients. Patients with high volume providers had slightly higher outpatient and drug costs (8% and 11%, respectively) than patients with lower volume providers.

## Discussion

The findings of this paper describe how medical complexity dramatically increases the already high baseline costs of care for PLWH. In our sample of Medicare enrollees with HIV, nearly 2/3 (64%) had at least one non-HIV related condition, and 39% had two or more. Mean costs of care increased from $30,312 for those without an identified comorbidity to over $46,000 for those with 2 comorbidities, and up to $219,000 for people with 11 or more. Our data confirm that the greater prevalence of comorbidities with increasing age of HIV-infected patients primarily accounts for increased inpatient and outpatient costs with age.[10]

The prevalence of comorbidities likely will increase with the aging of the PLWH population. For example, a modeling study in the Netherlands projects that the percentage of HIV-positive Dutch patients who are over 50 will increase from 28% in 2010 to 73% in 2030. The authors predict that 84% of Dutch HIV-infected patients in 2030 will have 3 or more non-communicable diseases—mostly cardiovascular.[25] Thus there is a need to better understand the impact of increasing comorbidities and in turn, increasing medical expenditures for treatment of PLWH. The current results, which show that including information on comorbidities improves the accuracy of predictions of median costs of treating PLWH, will allow for more accurate forecasts of future medical costs for an aging HIV population subject to greater numbers of comorbidities.

In the current analyses, pharmaceutical costs account for the greatest share of costs for PLWH, but are largely insensitive to comorbidities because the high cost of ART overshadows the cost of drug treatment of other comorbidities. Our data show that drugs account for a 64% of total average costs versus 18% for inpatient costs, continuing the pattern initiated when HAART first came into wide use.[18] In 1996, drugs accounted for 34% of total costs and inpatient 49% of total costs. By 1997, after wider adoption of HAART treatment, drugs’ share rose to over half of expenditures and the share of inpatient costs fell to 33% of a total cost of $1,521 per patient per month. [18,26] HAART improved overall survival of PLWH, resulting in extended lifetime, and greater prevalence of comorbidities in this population.

Evidence suggests that patients with HIV have greater numbers of health conditions than age-matched controls. [11,13] Consistent with this, among private insurance beneficiaries with pharmacy benefits, 53.6% of HIV-positive patients over 50 were prescribed more than 5 non-ARV medications over a 12 month period versus 34.3% of HIV negative patients of the same age group. [13]

In contrast to their moderate effect on pharmaceutical costs, comorbidities are associated with increases in median hospital and ambulatory care costs in the PLWH population. Historically, the development of ART dramatically lowered inpatient costs by reducing hospitalization rates for PLWH. Hospitalized PLWH are now more likely to die of conditions other than AIDS (e.g., non-AIDS infections, cardiovascular and liver disease) than of HIV-related conditions, having increased from 43% of hospital deaths among PLWH in 1995 to 70.5% of such deaths in 2011.[27] Our findings suggest that the impact of increasing comorbidities on costs is primarily through greater use of inpatient care, which often requires specialty procedures and intensive care.

This analysis is unique in looking at a range of non-HIV diagnoses to isolate the effect of each diagnosis on the resources used to treat PLWH. Our analysis examines a broad range of comorbidities, not just one, as in Yanik. [15] The most costly comorbidities occur less frequently and different comorbidities are diversely associated with each component of cost. Some, such as hemophilia and metastatic cancer, are associated with extremely high outpatient costs. Others, such as uncomplicated diabetes and pulmonary circulation disorders are associated with high costs for pharmaceuticals. Given the large share of spending on drugs, there is little opportunity to trim spending on either outpatient or inpatient services. [12, 28] An alternative strategy would be to reduce drug expenditures by negotiating with manufacturers to lower their prices—especially given the new recommendations to begin therapy immediately upon diagnosis.

Evidence of the relative contribution of lifestyle factors to morbidity among PLWH is beginning to accumulate.[29] Some have opined that lifestyle changes may have an even more important impact on health in HIV-infected individuals than in the general population, in part because they decrease the inflammatory state. [30] Indeed, we found that comorbidities relating to health habits–diabetes, hypertension, liver disease (hepatitis C), renal insufficiency, are prevalent among those infected with HIV.[2–9, 11] Thus, health related behavior change may be even more important for PLWH than for the general population. Yet, the ability of physicians to provide comprehensive preventive care and to monitor chronic conditions is constrained by relatively low Medicare and Medicaid reimbursement levels for outpatient primary care, particularly as compared with Ryan White reimbursements. Reimbursements for physician and outpatient clinics remain modest in US public insurance, and have been characterized as “quite meager and …inadequate to cover the cost of care provision at most HIV clinics in the United States, the majority of which are subsidized by federal and state dollars.”[31] Our finding that the share and level of outpatient expenditures are low for Medicare enrollees is particularly relevant because many former Ryan White clients, previously covered by a comprehensive fee, are now covered through the Medicaid expansion, at relatively low reimbursement rates.[32] Similar to Chen, [31] we conclude that outpatient reimbursements are low, given the high rates of preventable comorbidities and the presence of HIV treatment guidelines that call for a minimum of two visits and two viral load assays a year, as well as a number of immunizations and preventive health screens.

### Limitations

This paper has a number of limitations. First, the data relate only to California and may not generalize to other states. Secondly, only 20% of all PLWH have Medicare coverage. [33] Finally, the analyses rely on insurance claims data, so we do not observe services that were not submitted for reimbursement. However, providers had strong incentives to bill for services they provided. Thus we have confidence in the comprehensiveness of the reports.

## Conclusions

The aging of the population with HIV will increase the widespread prevalence of comorbidities among PLWH, adding to the already high costs of treatment for this population. We have shown that including comorbidity measures in predictions of future medical expenditures for PLWH will improve the accuracy of the spending forecasts. Many of the comorbidities that PLWH live with relate to potentially modifiable health habits. However, it may be difficult for providers to effectively provide interventions to modify negative health behaviors because of the small share of HIV care resources devoted to outpatient services.

## Abbreviations

AIDS: Acquired immune deficiency syndrome
ART: Antiretroviral therapy
CMS: U.S. Center for Medicare and Medicaid Services
HAART: Highly active antiretroviral therapy
HHS: U.S. Department of Health and Human Services
HIV: Human immunodeficiency virus
ICD-9: International Classification of Diseases *(*ICD*)*
MAE: Mean absolute error
PLWH: People living with HIV
RUCA: Rural-Urban Commuting Area Codes
